# Exploring the relationship between cyberbullying and unnatural child death: an ecological study of twenty-four European countries

**DOI:** 10.1186/1471-2431-14-195

**Published:** 2014-07-30

**Authors:** King-wa Fu, Chung-hong Chan, Patrick Ip

**Affiliations:** 1 Journalism and Media Studies Centre, The University of Hong Kong, Room 121, Eliot Hall, Pokfulam, Road, Hong Kong, SAR, China; 2 Department of Paediatrics and Adolescent Medicine, Li Ka Shing Faculty of Medicine, The University of Hong Kong, Hong Kong, SAR, China

**Keywords:** Child mortality, Europe, Internet, Cyberbullying, Statistics and numerical data

## Abstract

**Background:**

Internet risk has been recognised as a child safety problem, but evidence is insufficient to conclude that a child’s online risk exposure can lead to physical harm. This study aims to explore the ecological relationship between Internet risk exposure and unnatural child death.

**Methods:**

Multiple secondary data sources were used: online exposure to content about self-harm, cyberbullying, and Internet addiction data (EU Kids Online survey, 2010); and mortality data (European Detailed Mortality Database, 2010 or the latest year if not available) of 24 European countries. Correlations were found using quasi-Poisson regression. Countries’ prevalence rates of psychiatric problems (European Social Survey Round 3 and 6, 2006 and 2012) were used to test for possible spuriousness.

**Results:**

This study finds that countries with higher rates of cyberbullying were more likely to have a higher incidence of unnatural child death. A 1 percent rise in the prevalence of cyberbullying translated into a 28% increase in risk of unnatural child death (95% CI: 2%-57%). No evidence was found to substantiate confounding effect of the national prevalence of depressive symptoms or traditional bullying.

**Conclusions:**

Explanations are given for the findings. We conclude that intervention programs designed to serve as precautionary measures for risk minimisation should be considered.

## Background

While the Internet is considered as an essential platform through which the younger generation can learn effectively, participate in a variety of social and civic engagements, and interact with a broader spectrum of human activities [1], concern over younger people’s encounters with undesirable Internet content or risk is pervasive among health professionals, parents, and policy makers [2,3]. Broadly speaking, “Internet risk” is used as a collective term referring to the possibility of an unpleasant outcome, such as loss, injury, or harm, linked to an individual’s online exposure, which can be classified as online content, contact, and conduct [2]. For instance, exposure to suicide content on the Internet and cyberbullying may lead to suicidal behaviours, which cause public health concern [4,5]; Internet gaming addiction is included in Section III of the Diagnostic and Statistical Manual of Mental Disorders (DSM-5) as a condition that requires further research before it might become a new disorder [6]. Individuals with Internet addiction are often found to have depressive symptoms and suicidal ideation [7]. Young victims of online harassment have become increasingly prevalent [8], and such online experience has been found to be associated with their suicidal behaviours [9,10]. In the light of these findings, widespread concern has escalated over a potential “child safety” problem [2,3]. Guidelines have been developed by paediatricians to encourage parental control and intervention [11,12].

But an opposite view is held by social scientists who contend that the widespread concern over harmful consequences of online risk is merely a “moral panic” [13]. The critique draws heavily on the argument that despite a pervasive public anxiety, no empirical evidence has been established to support a real threat to a child’s physical safety literally caused by exposure to Internet risks, even though anecdotal evidence occasionally appears in the mass media [14]. Another consideration is that the Internet is only a secondary venue for manifest risk, whereas the victim of online harm may have been susceptible to other risk in the physical world, such as bullying or psychiatric problems [15], which may create a spurious relationship and thus confound the results.

In Europe, prevalence of Internet risks has been well documented [2,16]. For example, 7% of European children claimed to have experienced cyberbullying and 7% were exposed to “the websites that discuss ways of physically harming and hurting oneself” in the past twelve months [2]. In this study, we draw on secondary survey data and the mortality data sources collected from twenty-four European countries and examine the ecological relationship between Internet risk and unnatural child death.

## Method

### Data sources

Children’s Internet risk data were derived from the dataset released by the Economic and Social Data Service’s EU Kids Online survey (Study No. 6885, EUKOS) [17]. The EUKOS provides the data on Internet-related behaviours of children and parents in 25 European countries, including Austria, Belgium, Bulgaria, Cyprus, the Czech Republic, Denmark, Estonia, Finland, France, Germany, Greece, Hungary, Ireland, Italy, Lithuania, The Netherlands, Norway, Poland, Portugal, Romania, Slovenia, Spain, Sweden, Turkey, and the United Kingdom. The data were collected in a face-to-face survey in homes with Internet users aged 9 to 16 from the 25 countries; in all, 25,142 children were interviewed in 2010. The survey methodology and findings have been reported elsewhere [2,16]. Since another major data source in this study – the EDMD mortality database – only provides aggregated data in age groups 10–14 and 15–19 years, data samples of children aged 10–14, i.e. shared age range in both EUKOS and EDMD datasets, were selected for the analysis.

Four Internet risks were chosen and defined as follows:

1) Exposure to online information on self-harm or suicide: answering “Yes” to “Have you seen websites where people discuss ways of physically harming or hurting themselves or ways of committing suicide (either one)?”;

2) Experience of online and traditional bullying: answering “Yes” to “Has someone acted in this kind of hurtful or nasty way [this can include teasing someone in a way that the person did not like (online), hitting, kicking or pushing someone around, or leaving someone out of things (traditional)] to you on the Internet?”;

3) Experience of exclusively online bullying (excluding traditional bullying): respondent confirmed having had “the experience of online bullying” but answered “no” when asked if the experience had occurred “in person, face to face” and “by mobile phone calls, texts or image/video texts”;

4) Internet addiction (measured by a composite score): choosing an option among “very often”, “fairly often”, “not very often”, and “never/almost never” for a five-item scale: How often have the following things happened because of the Internet? 1) “Going without eating or sleeping”, 2) “feeling bothered when I cannot be on the Internet”, 3) “catching myself surfing when I'm not really interested”, 4) “spending less time than I should with either family, friends or doing schoolwork”, and 5) “trying unsuccessfully to spend less time on the Internet”.

The reference period for the above questions was twelve months. Missing responses were excluded from the analysis.

Based on each country’s data samples, prevalence rates of exposure to online information on self-harm or suicide, experiences of online and traditional bullying, experiences of exclusively online bullying, and the mean score of “Internet addiction” were calculated.

The European Detailed Mortality Database (EDMD) comprises the number of deaths for each European country, which are stratified by year, cause of death, age group, and sex [18]. The cause of death in EDMD was coded using the International Classification of Diseases (ICD-10), tenth revision [19], except for Greece. The sex and age stratified population figures of each country are also provided in the EDMD. Annual unnatural child death is defined as the collection of death causes including accidents (ICD-10: V01-X59), self-harm (ICD-10: X60-X84), assaults (ICD-10: X85-Y09), and undetermined cause of death (ICD-10: Y1-Y34) for children who died at age 10 to 14. For data from Greece, the cause of death is coded using ICD-9, the ninth revision [20]. The equivalent definition of unnatural death is devised as those who died from causes of death categorised with ICD-9 codes 800–999. For each country, the annual mortality incidence rate of unnatural deaths of children aged 10–14 was calculated (per 100,000).

Most countries’ mortality data were based on year 2010 figures, i.e. same year as the EUKOS study. When unavailable, the latest figures were used. Turkey’s data were not analysed because its death data were not available in the EDMD. Finally, data of twenty-four countries were included and analysed.

In the collected dataset, there were 1,013 unnatural child deaths (ages 10–14) among the twenty-four European countries. Of that total, 789 (77.9%) were accidental deaths, 138 (13.6%) were suicide deaths, 38 (3.8%) were caused by various forms of assault, and 48 (4.7%) were classified as undetermined causes of death. The unnatural death mortality incidence (ages 10–14) was ordered from the lowest 0 (Cyprus) to the highest 12.6 per 100,000 (Romania).

We also tested whether spurious associations of the national psychiatric problems might exist between Internet risk and unnatural causes of death, i.e. testing mediating and confounding effects using the same statistical procedure [21]. Proxy measures of a country’s prevalent rates of psychiatric problems were derived from the 2006 and 2012 European Social Survey respectively (ESS3 and ESS6) [22,23]. As the two datasets do not provide age breakdowns, respondents who were 18 years old or younger were selected as a proxy for each country. The depression symptom score was calculated using an eight-item version of the Center for Epidemiologic Studies Depression Scale Revised (8-item CES-D) [24]. It ranges from 0 to 24 and the clinical cut-off score is chosen as 7 [25]. It is interpreted as a higher score indicating a greater severity of depressive symptoms. Each country’s prevalence rates of depressive symptoms in 2006 and 2012 were computed to test for a possible spurious association respectively. Since some countries’ CES-D scores were not available in the two datasets, Czech Republic, Greece, Italy, Lithuania, and Romania were excluded in the 2006 spuriousness test. Austria, Greece, and Romania were excluded in the 2012 test.

The protocol of this secondary data analysis was approved by the Human Research Ethics Committee for Non-Clinical Faculties, The University of Hong Kong.

### Statistical analysis

The incidence of unnatural deaths was plotted against the prevalence rates of the four Internet risks and was analysed using quasi-Poisson regression [26]. The reason for using quasi-Poisson rather than conventional Poisson regression is to adjust for the spurious standard error due to the problem of dispersion. Negative binomial regression, which is also a common choice of over-dispersion adjustment, was not used because that technique gives higher weights to observations with a lower mean (that is to say, the countries with lower incidence of unnatural deaths), which may not provide a good estimate of the overall incidence of unnatural deaths for the current study [26].

The dependent variable was the raw count of unnatural deaths while the independent variable was the four Internet risks. The population difference among counties was adjusted by the introduction of an offset term of taking the logarithm of the age 10–14 population in the equation. Separate quasi-Poisson regression models were constructed for each Internet risks.

The Pearson’s correlations between the prevalence rates of depressive symptoms in 2006 and 2012 and the prevalence of exclusively online bullying were calculated. A quasi-Poisson regression model was also constructed such that unnatural cause of death was treated as a dependent variable, and the years 2006 and 2012 prevalence rates of depressive symptoms were treated as independent variables respectively. Statistical test of significance was set at the 5% level. We further deploy Cook's distance to quantify the effect of each data point on the model outcome [27]. An outlier is determined to be influential if its Cook’s distance is substantially larger than the rest [28].

## Results

**Figure 1:**
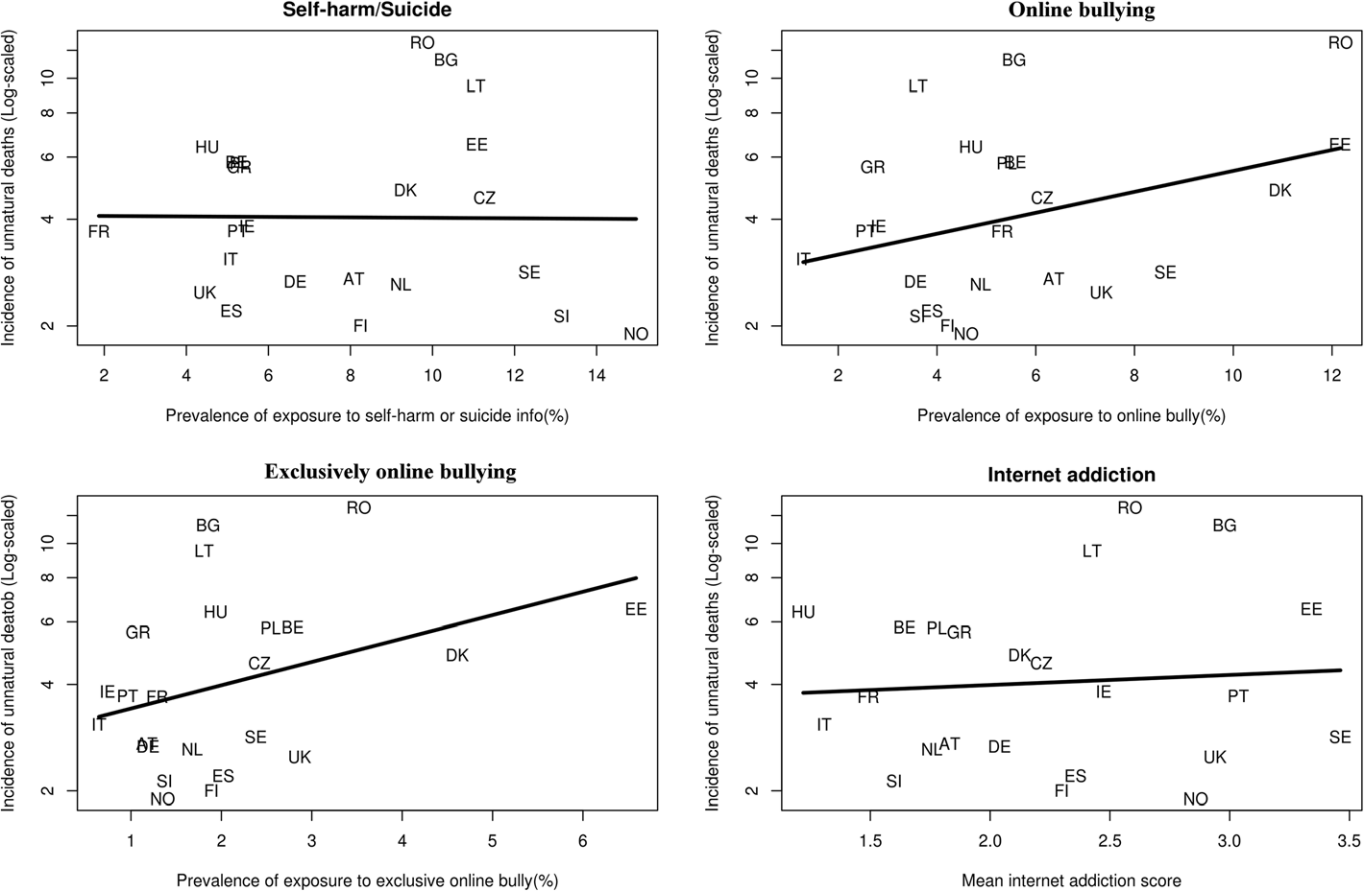
**Ecological associations between the incidence of unnatural child deaths (in log scale) and the four Internet risks.** Exploratory note: Each country’s incidence of unnatural deaths (y-axis in log scale) was plotted against its prevalence rates of the Internet risks (x-axis) in four separate panels respectively. Linear regression lines were fitted for easy inspection. Each data point represents a sampled country (See abbreviations).

The ecological associations between the incidence of unnatural child deaths and the four Internet risks are presented in Figure 1. The quasi-Poisson regression models are shown in Table 1. It confirmed that both the prevalence rates of online and traditional bullying and exclusively online bullying were both significantly associated with the mortality incidence of unnatural deaths of children in the twenty-four European country samples. The model also indicates that for a one percent increase in the prevalence of exclusively online bullying across a country, the risk ratio (relative risk) of unnatural deaths was expected to be e^0.244^ = 1.28 (95% CI: 1.02 to 1.57). For example, an increase in prevalence of exclusively online bullying from 5% to 6% between two countries represents an expected increase of annual incidence of unnatural child deaths from 6.48 to 8.28 per 100,000 people at risk (8.28/6.48 = 1.28).

**Table 1:** Regression analysis of the incidence of unnatural deaths among 10-14-year-olds in twenty-four European countries

	**Regression coefficient for intercept (SE)**	**Regression coefficient for exposure to risk (SE)**
Self-harm/suicide	−10.496 (0.284)**	0.058 (0.041)
Online bullying	−10.747 (0.232)**	0.116 (0.036)*
Exclusively online bullying	−10.623 (0.249)**	0.244 (0.109)*
Internet addiction	−10.196 (0.488)**	0.024 (0.225)

**Statistically significant, p < 0.001.

*Statistically significant, p < 0.05.

However, no significant correlations were found with the exposure to online information regarding self-harm or suicide and Internet addiction.

While controlling for the national prevalence rate of psychiatric problems, the prevalence of exclusively online bullying was not correlated with the national prevalence rate of depressive symptoms in 2006 (r = 0.213, p = 0.38) nor with the rate in 2012 (r = 0.085, p = 0.71). The quasi-Poisson regression analysis indicated that the prevalence rate of depressive symptoms in 2006 was not associated with the rate of unnatural child deaths (regression coefficient: −0.328, p = 0.78) and neither was the CES-D score in 2012 (regression coefficient: −1.553, p = 0.19). Since all spuriousness tests yielded nonsignificance, the spuriousness of the national prevalence rate of psychiatric problems is not substantiated.

Cook’s distance analysis found that the two most influential data points were Romania (Cook’s distance = 1.140) and the United Kingdom (Cook’s distance = 0.995), both of which had median high prevalence rates of exclusively cyber-bullying among all countries. The two countries with highest prevalence rates were Denmark (Cook’s distance = 0.041) and Estonia (Cook’s distance = 0.015). Since both values were considerably lower than those of Romania and the United Kingdom, we therefore concluded that the outlier data points had no substantial impact on the overall results.

## Discussion

To the authors’ knowledge, this paper reports the first study to explore the ecological relationship between the prevalence of Internet risks and the unnatural death of children in a country-level analysis. Even though the statistical power is limited, a statistically significant positive correlation between the prevalence of exclusively online bullying and unnatural-death mortality among children aged 10–14 was detected. This correlation is independent from the offline bullying because the correlation retained significance when those respondents who experienced offline bullying were excluded. This echoes a previous finding indicating that online bullying is a form of behaviour distinct from offline bullying [29], although another study suggests otherwise [30]. Moreover, no evidence is established to support a contention that a country’s prevalence of depressive symptoms confounds or mediates the observed ecological association. Even while low statistical power could be a reason for the insignificance, that lack of ecological correlation between variables cannot disprove the role of mental health conditions at the individual level.

Moreover, children’s exposure to suicide content and Internet addiction are found to have a statistically nonsignificant ecological correlation with unnatural child death rate. This may be a result of insufficient statistical power to detect small effect size or it may be because such correlations do not actually exist. Future research is needed to reexamine the question in a study design with sufficient power.

Previous work has suggested that experiencing online risk can result in negative emotions or self-harm [9]. However, general etiological theory behind the linkage remains unclear. It is believed to be multifactorial but the interplays between factors are subject to further investigation. There are a number of plausible explanations. One possible mechanism is that an individual’s mental health and well-being can be a mediator between Internet risk and unnatural death risk. Previous studies find adolescents involved in bullying are likely to develop mental health problems [31] associated with increasing risk of natural and unnatural causes of death (including accidental deaths) [32]. Internet risk, say cyberbullying, can escalate the risk of intentional injuries (deliberate self-harm or physical assault) or impaired mental health leading to increased risk behaviours, self-harm, impaired physical health, and consequently a higher likelihood of accidental and unnatural death. Another potential pathway is the linkage between poor mental health and well-being and exposure by some individuals to higher Internet risk, thus elevating the risk of unnatural causes of death as indicated in the previous study [32]. But it seems reasonable that these two mechanisms are not mutually exclusive. Each factor can reinforce the other and contribute to a lethal outcome. Moreover, there may be a reverse causation. An increase in suicide or unnatural death may arouse public interest and lead to increased attention and exposure to online content about suicide or cyberbullying.

Also, the observed association may be spurious due to unexamined ecological confounders. Examples could include health condition indicators such as prevalence rates of mental health disorders other than depression, alcoholism, or substance abuse, as well as country conditions such as household income, unemployment, social disparity, or economic development.

Caution should be taken when interpreting the findings because of some study limitations. As this study follows an ecological design, the results reflect only country-level correlations. We should avoid any claim regarding a correlation at the individual level [33]. Future research is warranted to assess the question using individual data, for example register-based or prospective cohort studies. Furthermore, secondary data analysis is used and therefore the data are not primarily collected for our study purpose and the data quality is out of our control. Another major limitation is the small sample size. Lack of statistical variability among countries may limit the study generalisation. The use of the CES-D is a proxy measure, and the prevalence rate of depressive symptoms may not entirely reflect a country’s mental health problems. Lastly, there were variations in the year of data collection among sampled countries.

Given these limitations and the exploratory nature of the study, some clinical implications are still worth noting. This study can generate useful hypotheses or research questions for future study, particularly for retesting the same hypothesis in non-European settings. Second, intervention and educational programs should be seriously considered as precautionary measures to minimise the chance of children’s experience of cyberbullying. Such proposed measures should be subject to rigorous effectiveness evaluation. Preliminary findings support restrictive parental control as an efficacious approach to reducing the likelihood of experiencing online risk [34]. Media literacy programs are suggested to mitigate the negative impact of Internet risk exposure on youth, but their effectiveness remains uncertain [35]. If future research consistently finds that some forms of Internet risk, for instance cyberbullying, can have an adverse universal impact on a country’s public health, policy making efforts and legislation to protect children’s online safety would be justified. Resources should also be allocated to fund and support intervention research and relevant studies focusing on policy and legal frameworks.

## Conclusion

This study reveals a positive ecological association between the rates of exclusively online bullying and unnatural-death mortality of the age 10–14 children among 24 European countries. It is also evident that this correlation is independent of the countries’ prevalence of depressive symptoms and traditional bullying. We therefore call for further research on this problem using individual study design and, for precautionary reasons, intervention programs for risk minimisation.

## Abbreviations

AT: Austria; BE: Belgium; BG: Bulgaria; CY: Cyprus; CZ: Czech Republic; DE: Germany; DK: Denmark; EE: Estonia; GR: Greece; ES: Spain; FI: Finland; FR: France; HU: Hungary; IE: Ireland; IT: Italy; LT: Lithuania; NL: Netherlands; NO: Norway; PL: Poland; PT: Portugal; RO: Romania; SE: Sweden; SI: Slovenia; TR: Turkey; UK: The United Kingdom.

## Competing interests

The authors declare that they have no competing interests.

## Authors’ contributions

KWF, CHC and PIP were involved in the study design and the analysis and interpretation of data. KWF and CHC were involved in drafting the article. All were involved in the final approval of the version to be published.

## Pre-publication history

The pre-publication history for this paper can be accessed here:

http://www.biomedcentral.com/1471-2431/14/195/prepub
